# Leptin and melatonin’s effects on OVX rodents’ bone metabolism

**DOI:** 10.3389/fendo.2023.1185476

**Published:** 2023-06-27

**Authors:** Zhenen Lin, Guanshu Yu, Shengren Xiong, Yu Lin, Zhaohui Li

**Affiliations:** Department of Orthopaedics, Fuzhou Second Hospital, School of Clinical Medicine, Fujian Medical University, Fuzhou, China

**Keywords:** Sema4d, leptin, melatonin, osteoporosis, bone metabolism

## Abstract

**Purpose:**

This study aims to examine the effects of leptin and melatonin intervention on bone metabolism in ovariectomize (OVX) rodents, as well as their potential mechanisms of action.

**Methods:**

Prepare an OVX model of osteoporosis in rodents and validate the model by collecting bilateral tibia samples for Micro-CT scanning and histological analysis. A control group of normal size, the OVX group, the OVX+Sema4D (Semaphorin 4D) group, the OVX+Sema4D+Leptin group, the OVX+Sema4D+ Melatonin(MT) group and the OVX+Sema4D+Leptin+ MT group were the experimental groups. Adenovirus vector construction and tibial medullary injection validation were conducted in accordance with the aforementioned experimental groups. Four groups of rats were injected with the Sema4D overexpression adenovirus vector into the tibial medullary cavity, and two groups were injected with the Leptin overexpression adenovirus vector. The repair of osteoporosis was observed using micro-CT and histological analysis. Immunohistochemical detection of bone morphogenetic protein-2 (BMP-2) expression in bone tissue was employed to ascertain the amount of osteoclasts in the upper tibial metaphysis, utilizing TRAP(tartrate-resistant acid phosphatase) staining.

**Results:**

Increased levels of BV/TV, Tb.N, BMD, and BMC were seen in the OVX+ Sema4D+Leptin, OVX+ Sema4D+MT, and OVX+ Sema4D+Leptin+ MT groups compared to the OVX group, whereas Tb. Sp levels were lowered. When compared to the Sema4D overexpression group, the trabecular bone structure of the OVX + Sema4D + Leptin, OVX + Sema4D + MT, and OVX + Sema4D + Leptin + MT groups is largely intact, tends to be closer, and the amount of trabecular bone increases. The OVX + Sema4D + Leptin + MT group in particular.The expression of BMP-2 was dramatically upregulated (p<0.05), the number of TRAP-stained osteoclasts was significantly reduced (p<0.05), and BALP(bone-derived alkaline phosphatase) and TRAP-5b(tartrate-resistant acid phosphatase-5b) activities were significantly downregulated (p<0.05).

**Conclusion:**

In rats with osteoporosis, leptin and melatonin can be seen to augment the trabecular microstructure of the bone, augment bone growth, diminish trabecular harm, and mend the bone. The combined effect is more powerful.

## 1 Introduction

Osteoporosis is a prevalent condition among middle-aged and geriatric individuals. Characterized by the decoupling of bone resorption and formation, bone reconstruction is characterized by a decrease in the number and activity of osteoblasts, and an increase in the number and activity of osteoclasts, leading to a reduction in bone mass per unit area and ultimately a decline in bone mineral density (BMD) and bone ageing (1). The current supposition is that a variety of genetic genes, environmental conditions, and body conditions control bone metabolism, with alterations in hormones, binding proteins, or other connections influencing bone metabolism being the primary determinants of bone tissue structure alterations (2–4). This is a highly sought-after subject in modern medical research.CD100, otherwise known as Semaphorin 4D, is a member of the signaling protein family first discovered in T lymphocytes and recently revealed to have a reduced part in bone regeneration. Although the bone density and thickness of Sema4D-deficient mice were greater than those of wild-type mice, it inhibited osteoblast differentiation *in vitro* by binding to PlexinB1 expressed on osteoblasts (5). In recent years, numerous cell experiments, animal experiments, and human studies have demonstrated that leptin and melatonin play a crucial role in modulating bone metabolism and in the process of bone reconstruction (6–8). In spite of this, research on how leptin and melatonin interact to control bone metabolism, prevent, and treat osteoporosis is still scant. Consequently, the combination of leptin and melatonin to reveal the inner relationship between them can offer fresh concepts and a theoretical basis for investigating the sources of osteoporosis and creating drugs for its prevention and treatment.

## 2 Materials and methods

### 2.1 Materials and reagents

SPF grade SD female rats, 200-220g, 7 to 8 weeks old, purchased from Zhejiang Weihua Experimental Animal Technology Co., LTD. Multifunctional enzyme marker, Shanghai flash spectrum; Primary antibody: BMP-2, Affinity; Leptin, Affinity; Sema4d, Cloud Clone; Secondary antibodies: Horseradish Enzyme-labeled Goat Anti-Rabbit IgG(H+L), Zhongshan Jinqiao; Tartrate-resistant acid phosphatase Staining Solution, Solarbio; Rat Tartrate-resistant acid phosphatase 5b, TRACP-5b ELISA, Jiangsu Meimian Industrial Co., Ltd; Rat Bone-derived Alkaline Phosphatase (BALP) Kit, Jiangsu Meimian Industrial Co., Ltd; Melatonin was purchased from Shanghai Yuanye Biotechnology Co., LTD. Sema4D overexpression plasmid and Leptin overexpression plasmid were synthesized by Anhui General Biology Co., LTD.

### 2.2 Experimental reagents and equipment

Pharmaceutical Refrigerator, Shandong Boke Biological Company; Reefer freezer, Midea; Slicer, Berner; Electric explosion Drying Oven, Shanghai Yuejin Medical Device Co., LTD. Olympus microscope and Leica slicing apparatus.

### 2.3 Construction of animal models and group administration

Animal experimentation’s goals and procedures are in accordance with accepted international norms and standards of morality. Prior to surgery, all rodents were fasted for 12 hours, isoflurane inhalation pre-anesthesia was administered 5 minutes beforehand, and continuous inhalation anesthesia was administered throughout the entire procedure. After administering anesthesia, the rodents were reclined on the rat plate, their abdominal hair removed, the area disinfected with iodophor, and a sterile towel applied. A longitudinal incision was made on the side of the abdomen, exposing the abdominal cavity and dividing it layer by layer. At the uterus’ extremity, the pale pink ovary encased in adipose tissue and the fallopian tube were visible. The fallopian tube was lapped using sutures, and ovarian tissue was removed using tissue forceps. After surgery, the peritoneum and skin were sutured in a sequence, the skin incisions were disinfected with iodophor, and the rodents were placed in a squirrel cage until they recovered. Three days later, they were observed and given an intramuscular injection of 20,000 units of penicillin G sodium to ward off infection. The model has been validated. Fifteen OVX rats were randomly divided into five distinct groups: OVX, OVX+Sema4D, OVX+Sema4D+Leptin, OVX+Sema4D+MT, and 5) OVX+Sema4D+MT +Leptin. Adenovirus injection: Rats were injected with Sema4D overexpressed adenovirus vector into the tibial pulp cavity in 4 groups, and Leptin overexpressed adenovirus vector was injected into the tibial pulp cavity in 2 groups. Before sampling, four days prior, rats were administered abdominal injection anesthesia, their lower limbs were shaved and disinfected, the patellar traction was opened along the knee joint to the side, the tibial plateau was exposed, a small hole was drilled in it, 50UL (titer 10^11) adenovirus was injected into the bone marrow cavity, and the hole was sealed with orthopedic paraffin wax. qPCR was used to detect Sema4D and Leptin in tibia tissue to confirm the efficiency of virus transfection. The fifth and sixth groups received intragastric administration of melatonin (50 mg/kg) once per day for 28 days, beginning in the eighth week of modelling.

### 2.4 A micro-CT system of high resolution was utilized to identify three-dimensional bone structure

One rat from each group was killed at the conclusion of the experiments, the soft tissues surrounding the tibia were removed under sterile conditions, the tibia underwent Micro-CT scanning and reconstruction (a unilateral tibia was taken, and a specific representative unilateral tibia was selected based on the results of model verification), and the six groups of indicators were then compared. Bone mineral content (BMC) (g), trabecular thickness (Tb.Th), number (Tb.N), distance (Th.Sp), and trabecular bone volume (BV/TV) (%); Bone mineral density (BMD) (g/cm3).

### 2.5 ELISA test

Centrifugation of the serum samples’ supernatant was conducted, and the BALP, TRAP-5b, and estradiol indices were then measured in accordance with the ELISA kit’s instructions. After returning all reagents and components to ambient temperature, the standard and sample are redrilled. Craft the operational answer for each element of the kit and read the reagents’ directions included with it. Remove the desired slats from the aluminum foil bag and place the remaining slats in a polythene bag in the refrigerator. Standard and sample wells are established. Standard well is supplemented with various concentrations of standard 50L. Set blank control holes and sample holes to be measured accordingly (blank control holes do not receive samples and enzyme-conjugate reagents; all other steps are identical).Gently agitate and mix the sample, avoiding contact with the well wall, by adding 40L to the enzyme label-coated sample dilution well, 100L to the well containing the sample to be tested, and the sample to the bottom of the well on the enzyme label plate. Add 100L of the enzyme-conjugated reagent to each well, excluding the blank well. Ensuring the plate is sealed with a film, incubating it at 37°C for a period of sixty minutes. For reserve use, the 20 times concentrated laundry solution is diluted 20 times with distilled water. With care, discard the film that seals the plate, then shake it dry. Fill each opening with a washable liquid, leave it for 30 seconds, and then discard. Do these five times, and pat the plate dry. In each well,50L of color developing agent A and then 50L of color developing agent B were added. The color developing process was initiated by shaking and stirring the mixture at 37°C for 15 minutes. To terminate the reaction (blue to yellow), add 50 L of termination solution to each well. The absorbance of each cavity was measured in succession at a wavelength of 450 nm. After the addition of the termination solution, the determination must be made within 15 minutes. After the detection is complete, the sample’s OD value is substituted into the equation to calculate the sample concentration, which is then multiplied by the dilution factor to acquire the sample's actual concentration.

### 2.6 HE staining

The tibia tissue was extracted and rinsed with water for three hours, then dehydrated with a 70%, 80%, and 90% ethanol blend, pure alcohol, and xylene blend for 15 minutes, followed by xylene I for 15 minutes, and xylene II for 15 minutes until transparent. Put the xylene and paraffin mixture in for 15 minutes, followed by paraffin I and paraffin II for 50-60 minutes each. Sectioning and embedding in paraffin. The paraffin segments are then dewaxed and hydrated after being baked. After being placed in distilled water, the sections were stained for 3 minutes in a hematoxylin aqueous solution, then differentiated by hydrochloric ethanol for 15 seconds, washed with a small amount of water, returned to the blue solution for 15 seconds, rinsed with running water, dyed with eosin for 3 minutes, rinsed with running water, dehydrated, transparent, sealed, and examined under a microscope.

### 2.7 TRAP coloring

Paraffin sections were dewaxed for five minutes and once more. Then, anhydrous ethanol for 5min,90% ethanol for 2min, and 70% ethanol for 2min, followed by natural drying and fixation with TRAP solution at 4°C for 30s-3min, or in most cases 30-60s.The slices were washed and lightly dried, then placed in TRAP incubation solution, placed in a 37°C temperature box, soaked for 45-60 minutes, washed with water, dyed with methyl green for 2-3 minutes, washed, dried, and examined under the microscope.

### 2.8 Immunohistochemistry

Two hours later, slice the tissue and bake it at 65°C.Submerged in xylene for 10 minutes, the slices were then placed in dewaxing for 10 minutes. Subsequently, for 5 minutes, the segments were immersed in 100% ethanol, 100% ethanol, 95% ethanol, 80% ethanol, and purified water. To repair the antigen, pepsin was used and incubated for 30 minutes at 37 degrees Celsius. To eradicate the endogenous peroxidase-blocking solution, the slices were placed in a moist box and treated with freshly prepared 3% hydrogen peroxide. After 10 minutes of incubation at room temperature, the segments were thoroughly washed with PBS. The transparencies were submerged in PBS three times for five minutes each time. The absorbent paper was used to dry the PBS around the tissue. The transparencies were coated with 5% BSA and sealed at 37°C for 30 minutes. Use primary antibody Absorb the sealing liquid surrounding the tissue with absorbent paper without rinsing, and add enough diluted primary antibody to each slide:BMP-2(1:200), Leptin (1:200), and sema4d (1:200) were deposited in a moist box and incubated at 4°C overnight. Remove the damp box, incubate at 4°C overnight, and employ the second antibody. Allow the slides to stand at room temperature for 45 minutes. Submerge the slides in PBS three times for five minutes each time. Under a microscope, the staining intensity was ascertained after 5-10 minutes of DAB coloring, followed by a rinsing of PBS or tap water for 1 minute. Hematoxylin was re-dyed for three minutes, hydrochloric acid alcohol differentiation, blue return; rinsing with tap water for one minute, dehydrating, sealing, and microscopy. The results of the staining were as follows: target protein, brown; nucleus, blue-purple.

### 2.9 Statistical analysis

Statistical analysis was conducted with SPSS 21.0 software, with quantitative results expressed by mean standard deviation (X SD). To compare two groups, an independent sample T test was employed, one-way analysis of variance was used to compare multiple groups, and the S-N-K method was used for pound-for-pound comparison. A test level of α=0.05, P<0.05, indicates a significant difference.

## 3 Results

### 3.1 Construction of an OVX model and verification of adenovirus transfection of osteoporosis in rats

**Figure 1:**
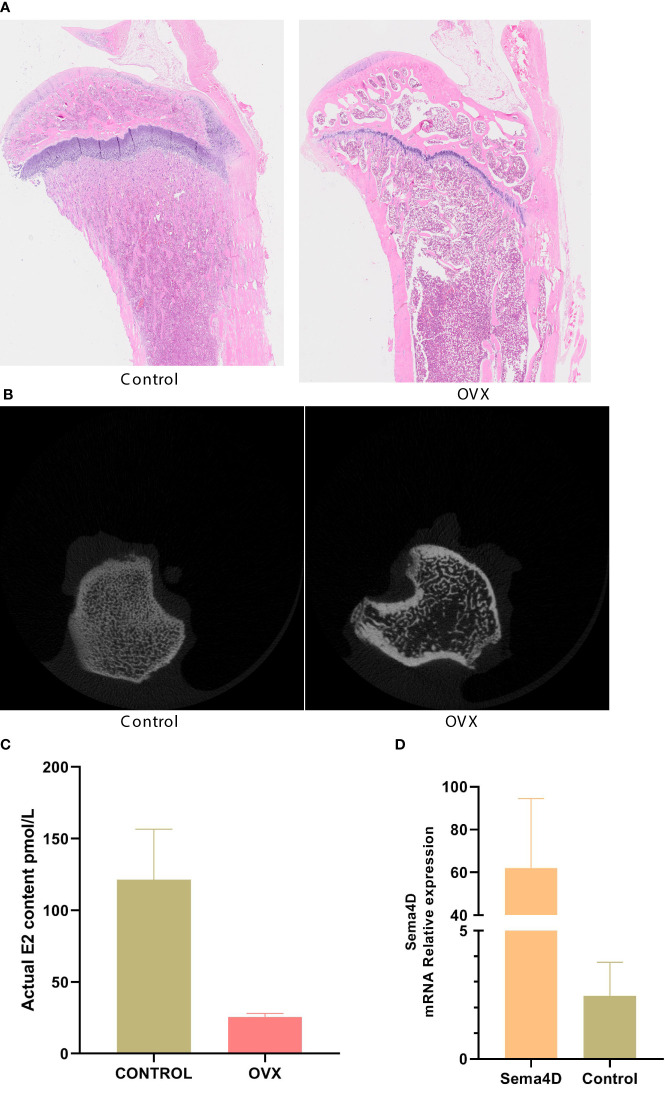
**(A)** Bone morphology was observed by HE staining; **(B)** Analysis of rat tibia specimens by Micro-CT scanning; **(C)** Serum estradiol was detected by ELISA; **(D)** The expression of Sema4D gene in tibia tissue was detected by qPCR to validate the efficacy of virus transfection. Compared to Control, p<0.05.

The results of HE staining, which was used to analyze bone morphology, showed that the control tibial bone trabeculae were densely packed and connected. However, the model group’s number of bone trabeculae drastically decreased, and the trabeculae became thinner, displaying a fracture shape (**Figure 1A**). Analysis of bilateral tibial specimens from rodents using micro-CT scanning revealed that the control group had a high number and density of bone trabeculae. The model group’s bone trabeculae both had a low number and density (**Figure 1B**). ELISA revealed variations in serum estradiol, and the results showed that, in comparison to the normal group, serum estradiol in the model group was significantly lower (**Figure 1C**), thus demonstrating the success of the model. To confirm the efficacy of virus transfection, qPCR was used to detect Sema4D gene expression in tibia tissue. The Sema4D expression group gene expression was found to be significantly greater than the control group, as demonstrated by the results (**Figure 1D**).

### 3.2 The effects of leptin and melatonin on tibial trabecular microstructure in mice with osteoporosis

**Figure 2:**
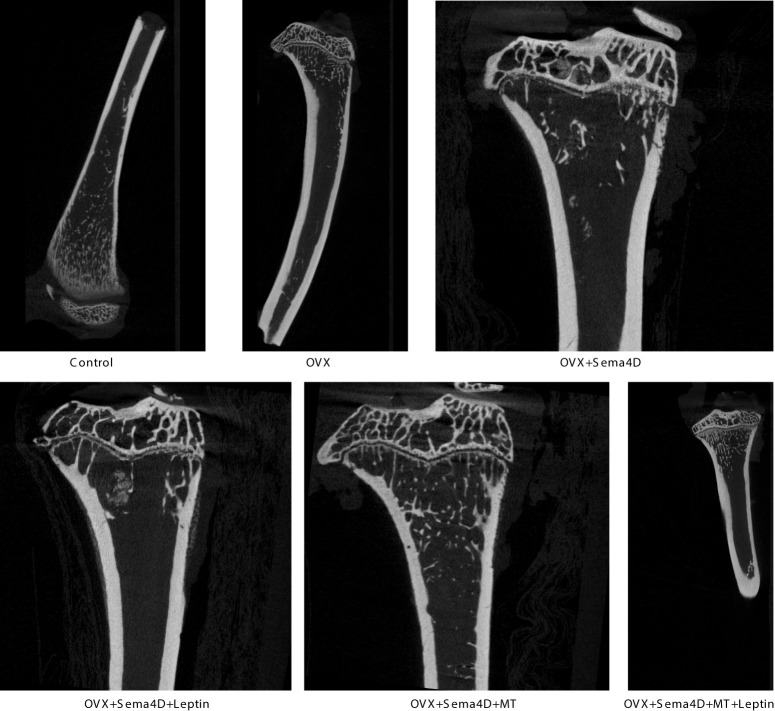
The tibia’s trabecular microstructure in rodents with osteoporosis is impacted by leptin and melatonin.

CT was used to detect bone structure, and the results showed that, compared to the OVX group, the levels of BV/TV, Tb.N, BMD, and BMC were increased, while the levels of Tb. Sp were decreased (**Figure 2**). **Table 1** shows that the OVX+ Sema4D+MT+Leptin group had the maximum levels of BV/TV, Tb.N, BMD, and BMC. The alteration of Tb.Th is not readily apparent.

**Table 1:** Trabecular bone microstructure values for each cohort.

Serial number	sample	BV/TV (%)	Tb.Th (mm)	Tb.N (1/mm)	Tb.Sp (mm)	BMD(g/cm^3^)	BMC (mg)
1	OVX	8.95452	0.10439	0.85782	0.71703	0.18658	0.46285
2	OVX+ Sema4D	4.40614	0.10878	0.40504	1.27876	0.13866	0.13876
3	OVX+ Sema4D+Leptin	8.36770	0.11393	0.73446	1.06630	0.17214	0.35939
4	OVX+Sema4D+MT	11.21440	0.08692	1.29018	0.46160	0.21645	0.83330
5	OVX+Sema4D+MT+Leptin	12.12530	0.11724	1.38208	0.39125	0.22364	0.94165

### 3.3 Observation of bone morphology of tibial tissue in rodents with osteoporosis by means of leptin and melatonin intervention

**Figure 3:**
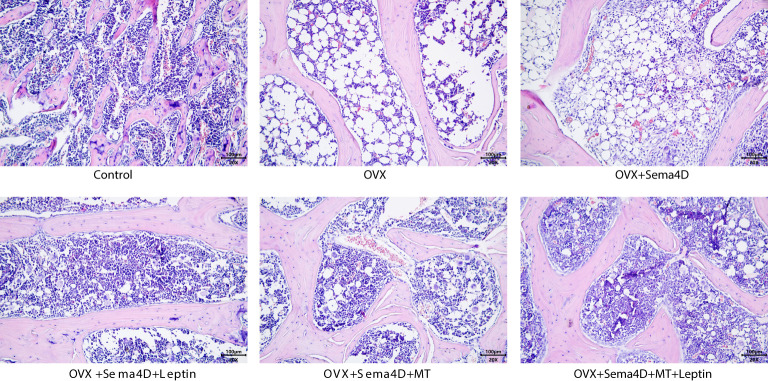
Observation of the bone morphology of tibial tissue in osteoporosis-affected rodents treated with leptin and melatonin.

HE staining was utilized to observe the alterations of tibia bone trabeculae. The control group’s bone trabeculae were found to be both stable and complete, while the OVX and OVX+Sema4D groups showed clear signs of harm to the trabecular structure, an irregular configuration, and a decrease in the number of trabeculae. The bone trabecular structure of the OVX+ Sema4D+Leptin, OVX+ Sema4D+MT, and OVX+ Sema4D+MT+ Leptin groups was comparatively complete, with a compact arrangement and a rise in the number of trabeculae (**Figure 3**) in comparison.

### 3.4 Effects of leptin and melatonin on osteoclast differentiation in osteoporotic rats’ tibial tissue

**Figure 4:**
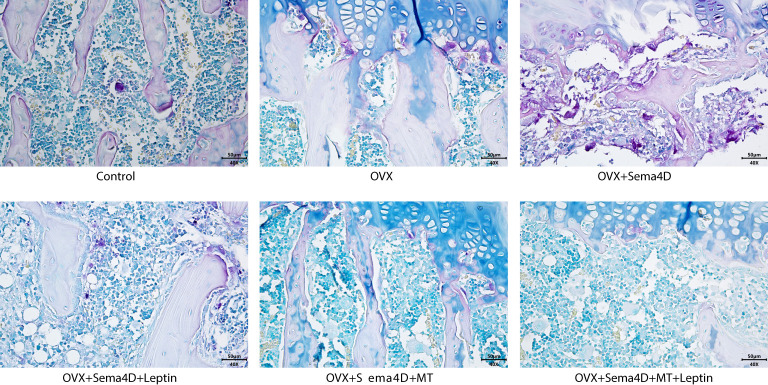
The differentiation of osteoclasts in rodents with osteoporosis, leptin and melatonin having a notable effect.

The upper tibial metaphysis was assessed using TRAP staining to ascertain the number of osteoclasts. In comparison to the control group, the OVX and OVX+Sema4D groups had a marked rise in TRAP-positive osteoclasts, with the greatest increase in the OVX+Sema4D group. In contrast, the OVX group, OVX+Sema4D+Leptin group, OVX+Sema4D+MT group, and OVX+Sema4D+MT+ Leptin group all had a considerable decrease in TRAP-positive osteoclasts (**Figure 4**) (see below).

### 3.5 Sema4d gene overexpression in conjunction with leptin overexpression or melatonin intervention on bone morphogenetic protein-2 in rodents with osteoporosis

**Figure 5:**
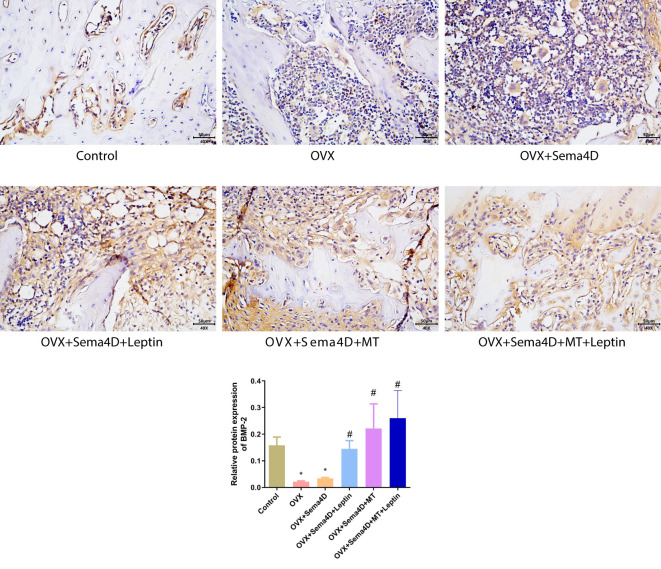
Immunohistochemical detection of BMP-2 protein expression and quantitative analysis in bone tissue is depicted. Compared to the Control group, *p<0.05; compared to the OVX group, ^#^p<0.05.

Immunohistochemistry was employed to ascertain the BMP-2 protein’s expression in tibia tissue, and the results showed a decrease in BMP-2 protein expression in the OVX and OVX+Sema4D groups when compared to the control group. In contrast, OVX, OVX+Sema4D+Leptin, OVX+Sema4D+MT, and OVX+Sema4D+MT+Leptin significantly augmented BMP-2 expression (**Figure 5**).

### 3.6 Leptin and melatonin intervention on BALP and TRAP-5b activities in osteoporosis rats

**Figure 6:**
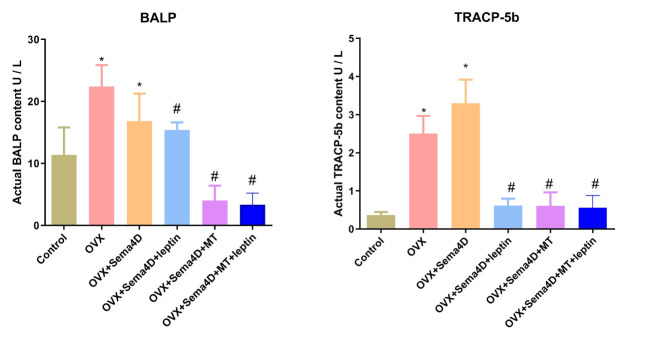
Leptin and melatonin activity on BALP and TRAP-5b in rodents with osteoporosis. Compared to the Control group, *p<0.05; compared to the OVX group, ^#^p<0.05.

The ELISA revealed that BALP and TRAP-5b activities were higher in the OVX group than in the control. However, BALP and TRAP-5b activities were lower in the OVX+ Sema4D+Leptin, OVX+ Sema4D+MT, and OVX+ Sema4D+MT+ Leptin groups than the OVX group (**Figure 6**) - a finding that was supported by the results.

## 4 Discussion

Aging brings about a disruption in the equilibrium between bone remodeling, resorption, and decoupling, which is the main source of osteoporosis. Leptin and melatonin are closely related to bone remodeling, which has become the focus of recent biological and medical research. A range of influencing elements, such as leptin and melatonin, control bone remodeling. Therefore, we intervene with leptin and melatonin. Currently, oophorectomy in rodents is a common technique for producing osteoporosis model rats with lower estrogen levels (9). We confirmed that both Leptin and MT can enhance and improve bone microstructure and metabolism in ovariectomized rats with osteoporosis by examining the tibia’s bone microstructure and metabolism. Further research revealed that the combination of Leptin and MT can also be beneficial. This could be a novel drug direction for osteoporosis prevention and clinical treatment.

The plexin family member, Plexin-B1, is expressed by osteoblasts, while its high-affinity ligand, semaphorin 4D (Sema4D), localizes to osteoclasts (10, 11). A 150-kDa glycoprotein transmembrane homodimer, Sema4D (CD100) - a member of the Semaphorin family - is a signaling protein implicated in axon-guiding processes. Recent data, however, suggest that Sema4D has additional functions, including immunomodulatory activity, platelet inactivation, angiogenic stimulation, and modulation of bone formation (5, 12, 13). The coupling factor Sema4D, expressed by osteoclast, has been proposed as a novel target for treating osteoporosis (14, 15). This gene has been found to have anabolic effects on bone, and its binding to PlexinB1, an osteoblast receptor, can inhibit its differentiation and function. Conversely, the disruption of Sema4D/PlexinB1 signal transduction, which activates osteoblast differentiation, can lead to an increase in bone loss (16, 17). In addition, Sema4D has been shown to be one of the hypoxia-efferent factors regulated by hypoxia-inducing factor (HIF-1) in a variety of cells, which plays a role in angiogenesis and generates bone protective properties through the circulatory system (18, 19). Therefore, the properties of Sema4D during bone remodeling can provide a new target for the discovery and development of anti-osteoporosis drugs. Our study also found that overexpression of Sema4D will aggravate osteoporosis, so further research on the related influencing factors of Sema4D expression is of great significance for the regulation of bone metabolism.

Leptin is a protein that is secreted and is implicated in insulin resistance, microvascular disease, inflammation, and osteoporosis. Recent studies have revealed that Leptin has a beneficial effect on bone synthesis, as it alters the differentiation of bone marrow mesenchymal stem cells into osteoblasts (20–22). In addition, Leptin can stimulate the central nervous system and/or neuroendocrine system to release corresponding mediators, thereby indirectly influencing bone metabolism (23, 24). However, it has also been determined that the effect of leptin signal changes on bone varies between axial bone, quadripartite bone, cortical bone, and trabecular bone, and that Leptin is primarily implicated in bone reconstruction in rats with bone loss due to ovarian resection. The number of osteoclasts in rats suffering from OVx-induced osteoporosis was significantly reduced by Leptin, while the expression of bone morphogenetic protein-2 and BALP/TRAP-5b activities were augmented. We therefore believe that Leptin has both a synthetic and direct regulatory influence on osteoclasts. Further investigation into the biological role of certain signaling pathways is still essential.

The pineal gland of mammals produces melatonin, an amine hormone with physiological roles such as regulating biological rhythms, anti-tumor, and anti-aging. Recent bone metabolism studies have demonstrated that melatonin can stimulate the proliferation and differentiation of osteoblasts, impede the proliferation and differentiation of osteoclasts, and foster the mineralization and maturation of bone matrix in a variety of ways. Bone marrow mesenchymal stem cells’ bone and lipid differentiation is regulated by it, thus making it a potential osteoporosis adjuvant drug (7, 25–27). By activating melatonin receptor 2(MT2), it has been discovered that MT can promote bone formation by up-regulating the expression of alkaline phosphatase (ALP), bone morphogenetic protein 2(BMP2), and other genes (28). Our research also demonstrated that MT can enhance bone microstructure and bone metabolism in rodents with oophorectomized osteoporosis. Despite the fact that numerous studies (29–32) suggest MT can reduce the harm caused by oxidative stress and inflammation to osteoblasts, as well as ward off osteolysis caused by reactive oxygen species and inflammatory factors, we still believe MT can be beneficial for the health of bone structure through its control of bone microenvironment and metabolism. Nevertheless, further research is needed for its clinical application in the prevention and treatment of osteoporosis.

Bone metabolism is uniquely impacted by leptin and melatonin. Further examination of the effects of LEP and MT on bone microstructure and bone metabolism in ovariectomized rodents with osteoporosis revealed that the combined use of LEP and MT can enhance and improve bone microstructure and bone metabolism, as well as inhibit the activation of Sema4d on osteoclasts. It is our conviction that LEP and MT, when combined, can not only impede the growth and activation of osteoclasts by controlling the activity of osteoblasts, but also directly obstruct the growth and activation of osteoclasts through distinct pathways to diminish osteolysis, thus making it a potential medication for the treatment of osteoporosis in the future. To better predict their potential anti-osteoporosis effects, it will be necessary in the future to investigate the specific mechanisms of different signaling pathways in promoting osteogenesis and inhibiting osteoclasts through cell experiments, animal experiments, and human studies.

## Data availability statement

The datasets presented in this study can be found in online repositories. The names of the repository/repositories and accession number(s) can be found in the article/supplementary material. Further requests can be sent to the authors.

## Ethics statement

The animal study was reviewed and approved by Animal Experiment Ethics Committee of Jiangxi Zhonghong Boyuan Biotechnology Co., Ltd. Written informed consent was obtained from the owners for the participation of their animals in this study.

## Author contributions

ZL designed the experiment and conducted the experiment. ZHL, SX helped ZL conduct some experiments and the experimental data is recorded. SX and YL instruct ZL to perform statistical analysis. GY wrote the paper, and ZL posted a comment, proposing appropriate revisions and corrections. All authors contributed to the article and approved the submitted version.

## References

[1] Martinis MD Sirufo MM Polsinelli M Placidi G Di Silvestre D Ginaldi L. Gender differences in osteoporosis: a single-center observational study. World J Mens Health (2021) 39(4):750–9. doi: 10.5534/wjmh.200099 PMC8443988 33474849

[2] Yao D Huang L Ke J Zhang M Xiao Q Zhu X. Bone metabolism regulation: implications for the treatment of bone diseases. Biomed Pharmacother (2020) 129:110494. doi: 10.1016/j.biopha.2020.110494 32887023

[3] Patil S Dang K Zhao X Gao Y Qian A. Role of LncRNAs and CircRNAs in bone metabolism and osteoporosis. Front Genet (2020) 11:584118. doi: 10.3389/fgene.2020.584118 33281877 PMC7691603

[4] Tencerova M Ferencakova M Kassem M. Bone marrow adipose tissue: role in bone remodeling and energy metabolism. best practice & research. Clin Endocrinol Metab (2021) 35(4):101545. doi: 10.1016/j.beem.2021.101545 33966979

[5] Shindo S Savitri IJ Ishii T Ikeda A Pierrelus R Heidari A. Dual-function semaphorin 4D released by platelets: suppression of osteoblastogenesis and promotion of osteoclastogenesis. Int J Mol Sci (2022) 23(6):1–6. doi: 10.3390/ijms23062938 PMC8955605 35328359

[6] Wee NKY de Lima TFC McGregor NE Walker EC Poulton IJ Blank M. Leptin receptor in osteocytes promotes cortical bone consolidation in female mice. J Of Endocrinol (2022) 255(1):25–37. doi: 10.1530/JOE-22-0084 35938692

[7] Guan H Kong N Tian R Cao R Liu G Li Y. Melatonin increases bone mass in normal, perimenopausal, and postmenopausal osteoporotic rats *via* the promotion of osteogenesis. J Trans Med (2022) 20(1):132. doi: 10.1186/s12967-022-03341-7 PMC8925213 35296324

[8] Kim B Kim YJ Kim JH Park K Ku H Choi Y-S. Melatonin protects bone microarchitecture against deterioration due to high-fat diet-induced obesity. J Bone Metab (2023) 30(1):69–75. doi: 10.11005/jbm.2023.30.1.69 36950842 PMC10036183

[9] Yousefzadeh N Kashfi K Jeddi S Ghasemi A. Ovariectomized rat model of osteoporosis: a practical guide. Excli J (2020) 19:89. doi: 10.17179/excli2019-1990 32038119 PMC7003643

[10] Vogler M Oleksy A Schulze S Fedorova M Kojonazarov B Nijjar S. An antagonistic monoclonal anti-Plexin-B1 antibody exerts therapeutic effects in mouse models of postmenopausal osteoporosis and multiple sclerosis. J Biol Chem (2022) 298(9):102265. doi: 10.1016/j.jbc.2022.102265 35850304 PMC9396414

[11] Kitamura K-I Takahira K Inari M Satoh Y Hayakawa K Tabuchi Y. Zebrafish scales respond differently to *in vitro* dynamic and static acceleration: analysis of interaction between osteoblasts and osteoclasts. Comp Biochem Physiol A-Molecular Integr Physiol (2013) 166(1):74–80. doi: 10.1016/j.cbpa.2013.04.023 23632157

[12] Jiang H Chen C Sun Q Wu J Qiu L Gao C. The role of semaphorin 4D in tumor development and angiogenesis in human breast cancer. Onco Targets Ther (2016) 9:5737–50. doi: 10.2147/OTT.S114708 PMC5045906 27729799

[13] Göbel A Kuhlmann JD Link T Wimberger P Link-Rachner C Thiele S. Plasma levels of semaphorin 4D are decreased by adjuvant tamoxifen but not aromatase inhibitor therapy in breast cancer patients. J Bone Oncol (2019) 16:100237. doi: 10.1016/j.jbo.2019.100237 31011525 PMC6461588

[14] Zhang Y Wei L Miron RJ Zhang Q Bian Z. Prevention of alveolar bone loss in an osteoporotic animal model *via* interference of semaphorin 4d. J Dent Res (2014) 93(11):1095–100. doi: 10.1177/0022034514552676 PMC4293773 25252878

[15] Anastasilakis AD Polyzos SA Makras P Gkiomisi A Sakellariou G Savvidis M. Circulating semaphorin-4D and plexin-B1 levels in postmenopausal women with low bone mass: the 3-month effect of zoledronic acid, denosumab or teriparatide treatment. Expert Opin Ther Tar (2015) 19(3):299–306. doi: 10.1517/14728222.2014.983078 25395071

[16] Negishi-Koga T Shinohara M Komatsu N Bito H Kodama T Friedel RH. Suppression of bone formation by osteoclastic expression of semaphorin 4D. Nat Med (2011) 17(11):1473–80. doi: 10.1038/nm.2489 22019888

[17] Lu L Huang J Zhang X Zhang J Zhang M Jing L. Changes of temporomandibular joint and semaphorin 4D/Plexin-B1 expression in a mouse model of incisor malocclusion. J Oral Facial Pain H (2014) 28(1):68–79. doi: 10.11607/jop.1082 24482790

[18] Qiu L Jiang H Luo J Xi J Wang X Pan Y. Regulatory sequence analysis of semaphorin 4D 5’ non-coding region. J Cancer (2019) 10(4):903–10. doi: 10.7150/jca.28169 PMC6400819 30854096

[19] Ding X Qiu L Zhang L Xi J Li D Huang X. The role of semaphorin 4D as a potential biomarker for antiangiogenic therapy in colorectal cancer. Onco Targets Ther (2016) 9:1189–204. doi: 10.2147/OTT.S98906 PMC4789851 27022279

[20] Li S Jiang H Wang B Gu M Zhang N Liang W. Effect of leptin on marrow adiposity in ovariectomized rabbits assessed by proton magnetic resonance spectroscopy. J Comput Assist Tomo (2018) 42(4):588–93. doi: 10.1097/RCT.0000000000000725 29489596

[21] Mei L Li M Zhang T. MicroRNA miR-874-3p inhibits osteoporosis by targeting leptin (LEP). Bioengineered (2021) 12(2):11756–67. doi: 10.1080/21655979.2021.2009618 PMC8810162 34818977

[22] Zheng B Jiang J Luo K Liu L Lin M Chen Y. Increased osteogenesis in osteoporotic bone marrow stromal cells by overexpression of leptin. Cell Tissue Res (2015) 361(3):845–56. doi: 10.1007/s00441-015-2167-y 25832621

[23] Fukuda T Takeda S. Secondary osteoporosis or secondary contributors to bone loss in fracture. regulation of bone homeostasis by nerve system. Clin Calcium (2013) 23(9):1279–83. 23999363

[24] Hipmair G Böhler N Maschek W Soriguer F Rojo-Martínez G Schimetta W. Serum leptin is correlated to high turnover in osteoporosis. Neuroendocrinol Lett (2010) 31(1):155–60. 20150868

[25] Zhang W-L Meng H-Z Yang R-F Yang M-W Sun G-H Liu J-H. Melatonin suppresses autophagy in type 2 diabetic osteoporosis. Oncotarget (2016) 7(32):52179–94. doi: 10.18632/oncotarget.10538 PMC5239543 27438148

[26] Tian Y Ming J. Melatonin inhibits osteoclastogenesis *via* RANKL/OPG suppression mediated by rev-erbα in osteoblasts. J Cell Mol Med (2022) 26(14):4032–47. doi: 10.1111/jcmm.17440 PMC9279587 35726597

[27] Huang X Chen W Gu C Liu H Hou M Qin W. Melatonin suppresses bone marrow adiposity in ovariectomized rats by rescuing the imbalance between osteogenesis and adipogenesis through SIRT1 activation. J Orthop Transl (2023) 38:84–97. doi: 10.1016/j.jot.2022.10.002 PMC9619141 36381247

[28] Li T Jiang S Lu C Yang W Yang Z Hu W. Melatonin: another avenue for treating osteoporosis? J Pineal Res (2019) 66(2):e12548. doi: 10.1111/jpi.12548 30597617

[29] Zhu F Liu Z Ren Y. Mechanism of melatonin combined with calcium carbonate on improving osteoporosis in aged rats. Exp Ther Med (2018) 16(1):192–6. doi: 10.3892/etm.2018.6141 PMC6030893 29977362

[30] Zhou L Chen X Yan J Li M Liu T Zhu C. Melatonin at pharmacological concentrations suppresses osteoclastogenesis *via* the attenuation of intracellular ROS. Osteoporosis Int (2017) 28(12):3325–37. doi: 10.1007/s00198-017-4127-8 PMC9841502 28956094

[31] Chen W Chen X Chen AC Shi Q Pan G Pei M. Melatonin restores the osteoporosis-impaired osteogenic potential of bone marrow mesenchymal stem cells by preserving SIRT1-mediated intracellular antioxidant properties. Free Radical Bio Med (2020) 146:92–106. doi: 10.1016/j.freeradbiomed.2019.10.412 31669348 PMC9805353

[32] Xu L Zhang L Wang Z Li C Li S Li L. Melatonin suppresses estrogen deficiency-induced osteoporosis and promotes osteoblastogenesis by inactivating the NLRP3 inflammasome. Calcified Tissue Int (2018) 103(4):400–10. doi: 10.1007/s00223-018-0428-y 29804160

